# Unveiling the molecule–plasmon interactions in surface-enhanced infrared absorption spectroscopy

**DOI:** 10.1093/nsr/nwaa054

**Published:** 2020-04-02

**Authors:** Jun Yi, En-Ming You, Song-Yuan Ding, Zhong-Qun Tian

**Affiliations:** State Key Laboratory of Physical Chemistry of Solid Surfaces (PCOSS), Collaborative Innovation Centre of Chemistry for Energy Materials (iChEM), and Department of Chemistry, College of Chemistry and Chemical Engineering, Xiamen University, Xiamen 361005, China; State Key Laboratory of Physical Chemistry of Solid Surfaces (PCOSS), Collaborative Innovation Centre of Chemistry for Energy Materials (iChEM), and Department of Chemistry, College of Chemistry and Chemical Engineering, Xiamen University, Xiamen 361005, China; State Key Laboratory of Physical Chemistry of Solid Surfaces (PCOSS), Collaborative Innovation Centre of Chemistry for Energy Materials (iChEM), and Department of Chemistry, College of Chemistry and Chemical Engineering, Xiamen University, Xiamen 361005, China; State Key Laboratory of Physical Chemistry of Solid Surfaces (PCOSS), Collaborative Innovation Centre of Chemistry for Energy Materials (iChEM), and Department of Chemistry, College of Chemistry and Chemical Engineering, Xiamen University, Xiamen 361005, China

**Keywords:** surface plasmons, surface-enhanced infrared absorption, Fano resonance, coupling strength, spectral lineshape

## Abstract

Nanostructure-based surface-enhanced infrared absorption (SEIRA) spectroscopy has attracted tremendous interest as an ultrasensitive detection tool that supplies chemical-fingerprint information. The interactions between molecular vibrations and plasmons lead to not only the enhancement of spectral intensity, but also the distortion of spectral Lorentzian lineshapes into asymmetric Fano-type or more complicated lineshapes in the SEIRA spectra; this effect hampers the correct readout of vibrational frequencies and intensities for an accurate interpretation of the measured spectra and quantitative analysis. In this work, we investigate the Fano interference between molecular vibrations and plasmons based on exact electrodynamic simulations and theoretical models. We report that, even if the molecular vibrational energy is equal to the plasmon resonant energy, the molecule–nanostructure distance-dependent dipole–dipole interactions, the plasmon-mediated coherent intermolecular interactions and the decay rates of plasmons have a significant impact on the SEIRA lineshapes. This study paves the way for controllable Fano interference at the nanoscale and more studies on plasmon-dressed molecular electronic or vibrational excited states.

## Teaser

From analyzing the spectral lineshape evolution in surface-enhanced infrared spectroscopy, we revealed how plasmons interacting with molecular vibrations at the nanoscale and predicted plasmon-mediated long-range intermolecular interaction.

## INTRODUCTION

Surface plasmon resonance refers to the light-driven collective oscillations of the conduction electrons of free-electron-like nanostructures, which confine the light into deep-subwavelength volumes (known as hotspots) with strongly enhanced optical fields [1]. When molecules are located at such hotspots, their interactions with light can be dramatically enhanced by orders of magnitude, leading to significantly magnified optical responses such as plasmon-enhanced Raman scattering [2–4], fluorescence [5], ultraviolet-visible absorption, infrared absorption [6–9] and non-linear vibrational spectroscopies [10,11]. Plasmon-enhanced spectroscopy techniques have become powerful tools for the identification and characterization of trace-molecule species with ultrahigh sensitivity down to the single-molecule level [12,13], leading to broad applications in chemical analysis and molecule-involved nano-optics [14].

However, the couplings between molecules and plasmons also inevitably induce strong modifications in the spectral lineshapes of the plasmon-enhanced spectra in addition to notably large enhancement in spectral intensities [15]. For example, the relative intensities of Raman bands in surface-enhanced Raman-scattering (SERS) spectra are found to be strongly distorted by the plasmonic background [16]; the surface-enhanced fluorescence (SEF) spectra of fluorescent molecules are also found to be tuned by plasmon resonant energy [17,18]. The surface-enhanced infrared absorption (SEIRA) spectra usually exhibit more complicated asymmetric Fano lineshapes [19–23]. Practically, it is difficult to quantitatively extract the intrinsic vibrational frequencies, bandwidth and absorption intensities of the probed adsorbates from the SEIRA spectra with complicated lineshapes, although the spectral resolution of the spectrometer itself is high enough, not to mention the quantitative analysis of mixing chemical compounds from the SEIRA spectra [24,25]. Therefore, it is important to reveal the mechanisms of these plasmon-tuned spectral shaping effects (PSSE), not only for better understanding of how molecules interact with plasmons at the nanoscale, but also for the extraction of the intrinsic chemical information of probed species, such as vibrational frequencies, molecular orientation, coverage, etc. from the plasmon-enhanced spectra.

The PSSE in SERS and SEF can be understood as the result of the plasmon-modified spontaneous emission rate and the spectral distortions to a large extent could be removed by normalizing the plasmon resonance spectroscopy [15,16]. For SEIRA, the asymmetric Fano lineshapes observed in the many SEIRA spectra characterize the feature of more complex interacting systems with Fano interference [19–21]. In typical Fano resonance, two different excitations interfere with each other. Specifically, one excitation is the transition to a discrete state and the other is the transition to a spectral continuum state [26–28]. In SEIRA, the Fano resonance might arise from interferences between the molecular vibrational excited state (taken as a discrete state) and plasmonic excited states (taken as a set of continuum states) [9]. The dependence of energy detuning (the energy difference between the intrinsic vibrational absorption energy and the energy at plasmon resonance) on the SEIRA lineshapes that evolve from a symmetric Lorentzian shape to an asymmetric Fano shape has been widely investigated [23,29]. Theoretically, a parameter-free model according to Fano's original quantum-mechanical derivations has been developed to successfully describe the detuning-dependent lineshape variations by considering the plasmonic resonance in the Fano model [24]. It has also been theoretically shown that the relative value of the external radiation loss and the intrinsic losses of the molecule–plasmon system could control the transition between electromagnetic-induced transparency with a Fano-dip lineshape and electromagnetic-induced absorption with an additional peak associated with the enhanced absorption [30]. However, beyond the detuning- and damping-dependent lineshape effect, the issue of how the molecule–plasmon near-field interactions directly control the evolutions of SEIRA spectral lineshapes has been rarely explored. Furthermore, beyond the two-body interaction picture, how the molecule–plasmon interactions for molecules with distinctive coupling strengths collectively control the evaluation of spectral lineshapes is also not clear.

Herein, we study the nanoscale couplings between the molecular vibrations and plasmons in a model system in the zero-detuning condition and study the coupling-strength-controlled evolutions of the SEIRA spectral lineshapes. Specifically, we investigate that the distance between molecules and the surface of the plasmonic nanostructure in the model system critically determines the molecule–plasmon coupling strength, and thus impacts the spectral lineshapes. Interestingly, a new mode occurs and red shifts if the molecular density increases and exceeds the critical density. The theoretical model studies reveal that this mode originates from the plasmon-mediated intermolecular coupling. Finally, the plasmon-decay-rate-controlled lineshapes are also discussed. This work unveils the mechanism of the asymmetry lineshape in SEIRA and paves the way for controllable Fano interference at the nanoscale.

## RESULTS AND DISCUSSION

**Figure 1.**
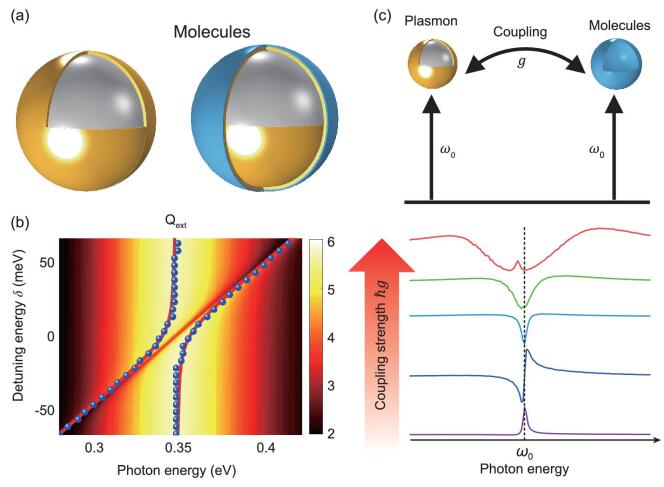
Schematics of the plasmon–molecule hybrid model system. (a) The model system consists of a Au nanoshell (SiO_2_@Au core–shell) and a molecular layer uniformly coating the surface of the nanoshell. The radius of the SiO_2_ core is 400 nm and the thicknesses of the Au shell and molecular layer are 6 and 1 nm, respectively. (b) Extinction efficiency spectra of the model system with various detuning energies. The blue dots indicate the energy of the hybrid modes extracted from the spectral peaks, which show apparent anti-crossing features. The split modes are fitted by a coupled harmonic-oscillators model, as shown in the red solid curves. The resonance energy of the nanoshell remains at 347 meV and the vibrational absorption energy of the molecules varies from 280 to 410 meV. (c) Energy diagram of the resonant molecule–plasmon coupling system. Optical transitions to vibrational or plasmonic modes are considered to be resonant with a frequency ${\omega _0}$. The typical absorption spectra of vibrational modes under various coupling strengths $\hbar g$ are shown in the bottom panel.

The plasmonic nanostructure studied in this work consists of a dielectric silica core and a thin metallic shell (SiO_2_@Au), as shown in Fig. 1a. Such core–shell nanoparticles were invented by the Halas group and are called nanoshells [31]. The SiO_2_@Au nanoparticles have been extensively studied for their plasmonic properties with tunable resonant wavelengths from the visible to mid-infrared spectral range [31] and in applications for SEIRA [8,32], SERS [32], solar energy [33], etc. The light-scattering problem of a concentric core–shell spherical particle can be analytically solved according to multi-layer Mie theory [34]. The resonant energy red shifts as the core becomes larger or as the shell becomes thinner (Supplementary Fig. 1). In this work, we chose a core size of 400 nm and a shell thickness of 6 nm as typical parameters for the nanoshell with a broad resonance band centered at${\rm{\ }}\hbar {\omega _{\rm{p}}}{\rm{\ = \ 0}}{\rm{.35\ eV}}$ (Supplementary Fig. 1b). The molecules were modeled by an ultra-thin (thickness of 1 nm) and uniform molecular layer (ML) covering the surface of the Au shell. The Lorentzian model is used to describe the optical absorption of the vibrational modes of the ML. The nanoshell and the molecular layer form a multi-layer particle (SiO_2_@Au@ML) as the model system for studies on the molecule–plasmon interaction and its impact on the lineshape of SEIRA spectra (see Supplementary Data 1 and Supplementary Fig. 2 for details).

The model system SiO_2_@Au@ML also exhibits energy-detuning-induced lineshape evolution. We studied the optical properties of the model systems with a fixed plasmon resonant energy $\hbar {\omega _{\rm{p}}} = \ 0.35\ {\rm{eV}}$ and with a tunable vibrational energy of the ML $\hbar {\omega _{\rm{m}}}$ that varied from 0.28 to 0.42 eV. The energy detuning is defined as ${\delta _{\rm{d}}} = \ \hbar {\omega _{\rm{m}}} - \hbar {\omega _{\rm{p}}}$. Figure 1b shows that the extinction spectra of the model system split into two modes for all detunings. Moreover, the detuning-dependent extinction spectra show an obvious mode ‘anti-crossing’ feature. By extracting the resonance energy of the two modes and further fitting the modes with the coupled-harmonic-oscillators model, we obtained the coupling strength $\hbar { g} = \ 8.05\ {\rm{meV}}$ of the molecule–plasmon coupled system [35] (see Supplementary Note 2 for details). The dispersion data of the coupled system are summarized in Supplementary Fig. 5. The fitted coupling strength of 8.05 meV is much smaller than the damping rate of the plasmons (with ${\gamma _{\rm{p}}} = \ 45.65\ {\rm{meV}}$), indicating that the coupling remains in the weak-coupling regime. In this regime, profound light-matter-interaction phenomena such as modified spontaneous emission (also known as the Purcell effect) and energy shift of the eigenstates (known as the Lamb shift) take place. To clearly show the modified SEIRA lineshape, we calculated the differential extinction spectrum by subtracting the extinction spectrum of the plasmonic structures (uncoupled system) from that of the molecule–plasmon coupling system, as shown in Supplementary Figs 2 and 3. For all detunings, the differential extinction spectra show Fano lineshapes, also indicating that the Fano interference emerges in the weak-coupling regime.

We introduce intuitive quantum-mechanical schematics to describe the coupling system, as shown in Fig. 1c and Supplementary Fig. 4. The molecular vibrational state is considered as a two-level system with a ground state $| 0 \rangle $ and an excited state $| e \rangle $ that interacts with the plasmon states $| P \rangle $. A new set of mixed eigenstates $| \Psi \rangle $ is formed due to the Fano interference between the molecules and plasmons. As a result, the optical responses of the coupling system are significantly modified. The typical absorption profiles as a function of the coupling strength are elaborated in Fig. 1c. In the weak-coupling region, the differential extinction spectra of the system are similar to the absorption of uncoupled molecules, indicating that the vibrational excited state is only slightly perturbed by the plasmon. When the molecule–plasmon coupling strength is reinforced, evolution of the Fano lineshapes of absorptions takes place, indicating that the molecular excited state becomes a significant optical dressing state. The system eventually enters the light-matter strong-coupling regime when the coupling strength $\hbar { g}$ exceeds the damping rate of the system, which is characterized by Rabi splitting of the modes. For the largest coupling strength, a new mode emerges in the spectra, as shown in Fig. 1c.

In this work, we introduce a theoretical model to describe the coupling system for better understanding of the underlying physics of the coupling-strength-dependent spectral shape [24,26,36]. A detailed derivation is given in the Supplementary Data 2. The absorption of the new eigenstates induced by the coupling between molecular vibrations and plasmons is given by the following:

(1) \begin{equation*}A( \omega ) = {I_{{\rm{in}}}} {C_{{\rm{ext}}}}( \omega )\frac{{{{( {q + \epsilon } )}^2}}}{{{\epsilon ^2} + 1}},\end{equation*}

where ${I_{{\rm{in}}}}$ is a constant parameter related to the incident intensity, ${C_{{\rm{ext}}}}( \omega )$ is the extinction cross section of uncoupled plasmonic structures, $\frac{{{{( {q + \epsilon } )}^2}}}{{{\epsilon ^2} + 1}}$ is a Fano function induced by the coupling [26] and the dimensionless parameters $q$ and $\epsilon $ are defined as follows:

(2) \begin{equation*}q\ = \frac{{{t_{\rm{m}}}}}{{\pi {\mu _{\rm{p}}}\sqrt {{\rho _{\rm{p}}}\hbar { g}} }}\ + \frac{{\hbar \omega - \hbar {\omega _{\rm{p}}}}}{{{\gamma _{\rm{p}}}/2}},\end{equation*}

(3) \begin{equation*}\epsilon \ = \frac{{\hbar \omega - \hbar {\omega _{\rm{m}}}}}{{\pi \hbar { g}}}\ - \frac{{\hbar \omega - \hbar {\omega _{\rm{p}}}}}{{{\gamma _{\rm{p}}}}}. \end{equation*}

In Eq. (2), ${\rho _{\rm{p}}}( {\boldsymbol{r}} )$ is the local density of photonic states such as the continuum plasmonic states, and ${t_{\rm{m}}}$ and ${\mu _{\rm{p}}}$ are the transition moments from the ground state to the vibrational excited states and to plasmonic states, respectively. Equations (2) and (3) also indicate that the Fano parameter $q$ critically depends on the coupling between the molecular vibrational excitation and plasmonic excitations, which relies on the near-field interactions.

**Figure 2.**
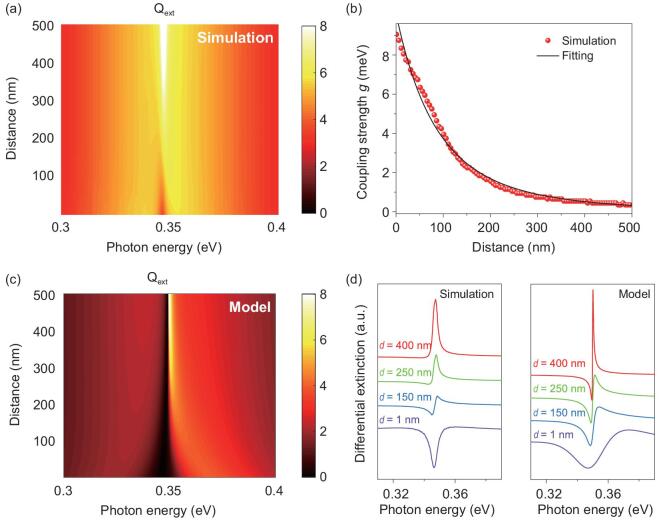
Distance-dependent spectral profiles. (a) Extinction-efficiency maps of the SiO_2_@Au@Air@ML hybrid system as a function of the thicknesses of the air layer $d$, which denotes the distance between the molecular layer (ML) and the surface of the Au shell. The thicknesses of the Au shell and ML are fixed. (b) Coupling strength $\hbar g$ as a function of the separation distance. For the zero-detuning case (resonant coupling), the coupling strength is equal to half of the Rabi splitting. The coupling strength is further fitted by an analytical model, with the coupling strength exponentially decaying as a function of the distance. The result shows that $\hbar g$ is proportional to ${d^{ - 6}}$. (c) Extinction-efficiency maps based on the theoretical model, as shown in Eq. (1). The coupling strength is extracted from Fig. 2b. (d) Typical differential extinction spectra of molecules with various separation distances $d$ from the exact simulation (left panel) and from the theoretical model (right panel).

The plasmonic near-field distribution is spatially inhomogeneous and the field intensity exponentially decays away from the surface of the plasmonic structure. As a result, the lineshape of the absorption spectra should be dynamically changed if the molecule–nanoshell distance changes. We consider a model structure SiO_2_@Au@Air@ML with the air layer in between the Au shell and molecular layer to study the extinction spectra map as a function of molecule–nanoshell separation distance $d$ under the zero-detuning condition (${\delta _{\rm{d}}} = \ \hbar {\omega _{\rm{m}}} - \hbar {\omega _{\rm{p}}} = \ 0$), as shown in Fig. 2a. For $d$*<* 80* *nm, the spectra show a dip feature near 0.347 eV, which is the resonance energy of the uncoupled vibration and plasmonic structure. For larger $d$ varying in the range 100–200 nm, the dip evolves to an asymmetric Fano shape. The spectra eventually change to Lorentzian-like resonance bands for $d$ > 250 nm. The $d$-dependent spectral lineshape evolution can be understood according to Eqs (1)–(3) as a result of the decrease in coupling strength. Under the zero-detuning (${\rm{\omega }} \to {\rm{\ }}{\omega _{\rm{m}}} = {\omega _{\rm{p}}}\ $) condition, the asymmetry factor $q$ is inversely dependent on the square root of the coupling strength. For small $d$, $q$ approaches zero and the absorption intensity is roughly proportional to ${q^2}$ as a result of the maximal coupling strength (∼16 meV). As $d$ increases, the coupling strength exponentially attenuates, followed by a rapid increase in $q$, and thus the spectra evolve to an asymmetric Fano shape and eventually to Lorentzian-like shapes when $q$ approaches infinity.

We extracted the coupling strength $\hbar { g}$ as a function of $d$ and found that the function was well fitted with the expression ${ g}\sim 1/{d^6}$, as shown in Fig. 2b, which indicates that the origin of the coupling is mainly dominated by the dipole–dipole interactions between the molecular vibrations and the plasmons. Interestingly, the profile of the differential extinction spectra can also be theoretically predicted by Eqs (1)–(3), where the transition parameters ${\mu _{\rm{p}}}$ and ${t_{\rm{m}}}$ are approximately estimated via the parameter-free approach [24] (see Supplementary Note 3 for details). As shown in Fig. 2c and d, the theoretical results satisfactorily reproduced the main features such as the spectral lineshapes and intensities of the evolution spectra calculated by exact electrodynamic simulations, according to the multi-layer Mie theory. Our model also predicts that the spectral bandwidths become narrower at a large distance because the decay rate of the hybrid states is linearly dependent on the coupling strength. We note that the theoretical-predicted lineshape slightly differs from the simulation results for larger $d$ because the coupling strength depends on the local density of the photonic states ${\rho _{\rm{p}}}( {\boldsymbol{r}} )$, which is spatially varied and was not considered in the theoretical model.

In reality, the molecules are spatially distributed on the surface of the plasmonic structures. The optical responses of the multilayered particle system thus undergo complex contributions from the molecule–plasmon coupling with molecular-position-dependent coupling strength $\hbar { g}( {\boldsymbol{r}} )$. However, the overall optical response of the hybrid system is the coherent superposition of all interactions, instead of the simple sum of the absorption spectrum from each molecule. In this scenario, all molecules are indirectly coupled via the coherent plasmonic field and behave in the manner of a delocalized macromolecule with notably large coupling strength, thus transforming the response into the collective behaviors of the entire molecular ensemble. This plasmon-mediated long-range intermolecular interaction triggers striking physical phenomena and applications in the framework of cavity quantum electrodynamics (CQED) [37], such as cavity-mediated long-range energy transfer [38,39] and charge transfer [40]. In the following, we show that such a coherent interaction leads to an emergent mode in the SEIRA spectra.

**Figure 3.**
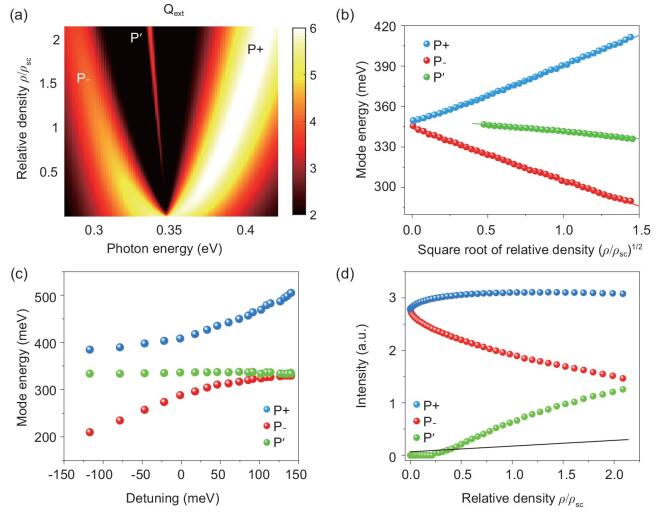
Molecular density-dependent coupling strength and spectral profiles. (a) Simulated extinction efficiency of the hybrid system with various relative molecular densities $\rho $. The ${\rho _{{\rm{sc}}}}$ denotes the critical density to reach the strong-coupling regime. (b) Extracted-mode energy as a function of the square root of the relative density. The shift of the P– and P+ modes shows linear dependency on the square root of the relative density, which is predicted by the Tavis-Cummings model. (c) Detuning-dependent-mode dispersion of the hybrid system with $\rho /{\rho _{{\rm{sc}}}} = 2$ (Rabi splitting ∼120 meV). The new P^′^ mode minimally changes with detuning. (d) Extracted extinction peak intensity as a function of the relative density. All three modes show the non-linear dependence of the extinction intensity on${\rm{\ }}\rho /{\rho _{{\rm{sc}}}}$. In contrast, the absorption intensity of a pure molecular layer without plasmonic structures shows linear dependence (black solid line).

We consider the multi-layer particle (SiO_2_@Au@ML) with increased oscillation strength of the molecules, which is thus equivalent to an increase in the molecular density $\rho $. Figure 3a shows that the extinction spectra dramatically evolve as the density gradually increases under the zero-detuning condition (see Supplementary Note 1 for details). For low density $\rho {\rm{\ }}\sim{\rm{\ }}0$, the spectra display Lorentzian-like profiles as a result of weak coupling between the molecular vibration and plasmons. As the density increases, the dip features emerge and thus the spectra show two split branches labeled as P– with lower energy and P+ with higher energy. Moreover, the energy splitting between P– and P+ increases as the density $\rho $ increases. The energy splitting exceeds the decay rate of the plasmons and the coupled system thus enters the strong-coupling regime [41] when the density approaches the critical threshold ${\rho _{{\rm{sc}}}}$. Interestingly, a new resonant mode labeled as P^′^ emerges when $\rho /{\rho _{{\rm{sc}}}}$ exceeds 0.23 and the P^′^ mode slightly red shifts as $\rho /{\rho _{{\rm{sc}}}}$ further increases. The detailed spectra progressions are shown in Supplementary Fig. 6.

Figure 3b summarizes the resonant energy of the modes as a function of the relative density $\sqrt {\rho /{\rho _{\rm{sc}}}}$. We found that the energy splitting between P– and P+ scales up with the square root of the density *ρ* in that $\hbar {\rm{\Omega \ }} = {\rm{\ }}( {\hbar {\omega _{P + }} - \hbar {\omega _{P - }}} ) \propto \sqrt \rho $. The $\sqrt \rho $ scale law lies in the coherent molecular vibrations. Additionally, the intermolecular interaction is not included in the Mie-theory model. Thus, the $\sqrt \rho $ scale law indicates the collective molecule–plasmon couplings in our system [37,39]. This scenario is equivalent to multiple identical two-level emitters interacting with a plasmonic mode, which is depicted by the Tavis-Cummings model in the full quantum picture [42]. We note that the similar behaviors of the modes are also observed in the exciton–plasmon interacting system in the visible spectral range, since the dielectric descriptions of molecular excitonic and vibrational excitations are similar. However, the resulted energy shift of the P^′^ mode could be different for the exciton case and molecular-vibrations case [43,44].

To understand the origin of the P^′^ mode, we analysed the detuning dispersion of the three modes under the condition of a high molecular density, e.g. $\rho /{\rho _{{\rm{sc}}}} = \ 2$, by tuning the plasmon energy (through changing the thickness of the metal shell) and fixing the molecular vibrational energy. The detailed extinction maps with various detuning energies are shown in Supplementary Fig. 7 and three modes are observed. In Fig. 3c, we plotted the peak energies extracted from Supplementary Fig. 7 to analyse the dispersions. The P+ and P– modes (blue and red dots, respectively) show anti-crossing features with Rabi splitting of 120 meV. The new P^′^ mode (green dots) is minimally dispersed as the plasmon energy is tuned and slightly red shifts (∼10 meV), indicating the molecular vibrational nature of P^′^ mode rather than a plasmonic character. One might intuitively consider P^′^ mode as a result of the intrinsic uncoupled molecules. However, the P^′^ mode behaves distinctly differently from the uncoupled molecular absorption. As shown in Fig. 3d, we summarized the extinction intensity variation as a function of $\rho /{\rho _{{\rm{sc}}}}$. The extinction intensity of the SiO_2_@ML core–shell structure without the Au nanoshell (black solid line in Fig. 3d) linearly depends on $\rho /{\rho _{{\rm{sc}}}}$, as expected by excluding the plasmon. However, all of the P–, P+ and P^′^ modes show non-linear dependence on the density if the plasmon is included. Specifically, the extinction intensity of the P– mode gradually decreases as the density increases, whereas the P+ mode increases in the extinction intensity and quickly approaches saturation. Interestingly, the P^′^ mode appears only if the density exceeds a certain threshold and undergoes a sublinear increase with the density, as shown in the dotted curves in Fig. 3d, which is significantly deviated from the linear behavior when plasmon is excluded. The distinct non-linear dependency confirms that the P^′^ mode is not the optical response from the uncoupled molecules, instead indicating the plasmon-mediated coherent intermolecular-interaction effect.

**Figure 4.**
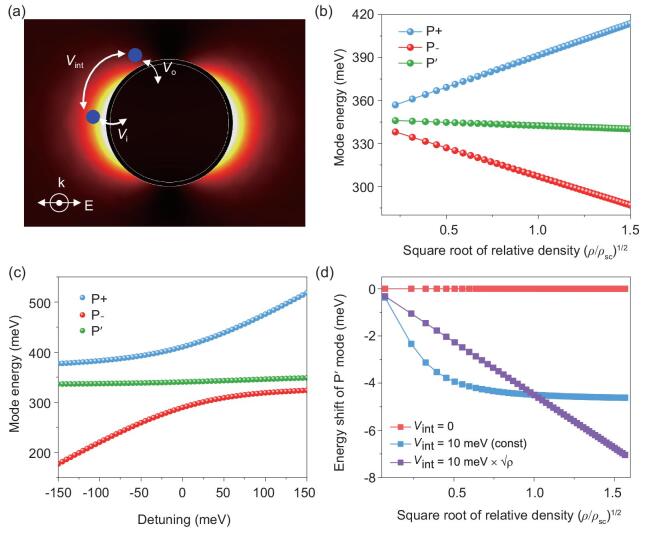
Analysis of the modes based on coupled harmonic-oscillator models. (a) The schematics of plasmon-mediated intermolecular interaction. Herein, the molecules (blue dots) located inside the hotspot and outside the hotspot couple with plasmons with different coupling strengths (${V_{\rm{i}}}$ and ${V_{\rm{o}}}$). The molecules at the two locations are indirectly coupled through the plasmonic field and the phenomenal interaction strength is ${V_{{\rm{int}}}}$. (b) The fitted-mode energy as a function of the square root of the relative molecular density. The parameters used in the fitting are $\hbar {\omega _{{\rm{m}},{\rm{out}}}} = \ \hbar {\omega _{{\rm{m}},{\rm{in}}}} = \ \hbar {\omega _{\rm{p}}} = \ 0.347\ {\rm{eV}},$${V_{{\rm{inter}}}} = \ {V_{{\rm{out}}}} = \ 10\ {\rm{meV}}$ and ${V_{{\rm{in}}}} = \ 40\ {\rm{meV}}$. (c) Detuning-dependent-mode dispersion of the hybrid system with *ρ* = 2 (splitting of P+ and P– mode ∼120 meV). Here, the plasmon resonance energy $\hbar {\omega _p}$ increases from 0.15 to 0.6 eV, while the molecular resonance $\hbar {\omega _{{\rm{m}},{\rm{out}}}},{\rm{\ }}\hbar {\omega _{{\rm{m}},{\rm{in}}}}$ and the interaction strength are kept the same as those used in panel (b). (d) Energy shift of the P^′^ mode in different assumptions as a function of the square root of the relative molecular density. Here, we calculated the energy shift of the P^′^ mode (away from $\hbar {\omega _{{\rm{m}},{\rm{in}}}} = \ 0.347\ {\rm{eV}})$. ${V_{{\rm{out}}}}$ is 10 meV and ${V_{\rm in}}$ is 40 meV. For ${V_{{\rm{inter}}}}$= 0 (red curve), i.e. the plasmon-mediated intermolecular interaction is excluded, the energy of the P^′^ mode will be exactly fixed at 0.347 eV. If ${V_{{\rm{int}}}}$ is a constant that does not scale up with the molecular density (blue curve; here, we set ${V_{{\rm{int}}}} = {V_{{\rm{inter}}}}\ $= 10 meV), the P^′^ mode first nonlinearly red shifts and then approaches to a constant at higher density. For the $\ {V_{{\rm{int}}}} = \ {V_{{\rm{inter}}}}\sqrt \rho $ case (purple curve; here, we set ${V_{{\rm{inter}}}}$ = 10 meV), the P^′^ mode linearly red shifts as $\sqrt \rho $ increases, which reproduces the simulation results.

In the strong-coupling regime, the previously proposed Fano formula become invalid as the molecular vibrations and plasmons are strongly hybridized, forming the so-called vibro-polaritons [37,45]. Thus, we introduce the coupled harmonic-oscillators model that involves the plasmon-induced intermolecular interaction to describe the hybrid modes (see Supplementary Note 2). Generally, the molecules in the molecular shell locate at various positions such as inside the hotspots and outside the hotspots for simplicity and experience different coupling strength with the plasmons. Thus, the molecules inside the hotspot and those outside the hotspots can be simplified as two kinds of oscillators that coherently coupled with plasmon with distinctive coupling strength: the ${V_{\rm{i}}}$ and ${V_{\rm{o}}}$ terms, respectively, as shown in Fig. 4a. Due to the coherent coupling, the molecules at the two locations are indirectly coupled through the plasmonic field and phenomenally leading to long-range intermolecular interactions that are mediated by plasmons (${V_{{\rm{int}}}}$ term). The Hamiltonian matrix of the interacting system, ${{\bf H}}$, is given by

(4) \begin{equation*}{{\bf H}}\ = \left( {\begin{array}{@{}*{3}{c}@{}} {\hbar {\omega _{{\rm{m}},{\rm{out}}}}}&{{V_{{\rm{int}}}}}&{{V_{\rm{o}}}}\\ {{V_{{\rm{int}}}}}&{\hbar {\omega _{{\rm{m}},{\rm{in}}}}}&{{V_{\rm{i}}}}\\ {{V_{\rm{o}}}}&{{V_{\rm{i}}}}&{\hbar {\omega _{\rm{p}}}} \end{array}} \right),\end{equation*}

where $\hbar {\omega _{{\rm{m}},{\rm{out}}}}$, $\hbar {\omega _{{\rm{m}},{\rm{in}}}}$ and $\hbar {\omega _{\rm{p}}}$ are the vibrational energies of the molecules outside the hotspots and inside the hotspots, and the resonance energy of the plasmonic structure, respectively. Under the zero-detuning condition, $\hbar {\omega _{{\rm{m}},{\rm{out}}}} = \ \hbar {\omega _{{\rm{m}},{\rm{in}}}} = \ \hbar {\omega _{{\rm{m}},{\rm{p}}}}$. ${V_{{\rm{int}}}}$, ${V_{\rm{o}}}$ and ${V_{\rm{i}}}$ are the plasmon-mediated intermolecular coupling strength, the molecule–plasmon coupling strength in the molecules outside the hotspots and that of the molecules inside the hotspots, respectively. The molecule–plasmon coupling strength ${V_{\rm{i}}}$ and ${V_{\rm{o}}}$ would scale up with the square root of the molecular density$\sqrt \rho $, as elaborated previously. Here, we define ${V_{\rm{i}}} \equiv {V_{{\rm{in}}}}\sqrt \rho $ and ${V_{\rm{o}}} \equiv {V_{{\rm{out}}}}\sqrt \rho $ to clarify the scaling law. Since the coupling strength depends on the local density of the photonic states, as we discussed above, it thus requires ${V_{{\rm{in}}}} > {V_{{\rm{out}}}}$. For the plasmon-mediated intermolecular coupling term ${V_{{\rm{int}}}}$, we phenomenologically define ${V_{{\rm{int}}}} \equiv {V_{{\rm{inter}}}}\sqrt \rho $ by assuming that ${V_{{\rm{int}}}}$ scales up with $\sqrt \rho $, since the plasmon-mediated intermolecular interaction is dominated by the interactions between the plasmon and the molecules outside the hotspots. This point will be further verified as elaborated below.

Using the parameters from the simulations as $\hbar {\omega _{{\rm{m}}{,}{\rm{out}}}} = \ \hbar {\omega _{{\rm{m}}{,}{\rm{in}}}} = \ \hbar {\omega _{\rm{p}}} = \ 0.347\ {\rm{eV}}$ and assuming ${V_{{\rm{inter}}}} = {V_{{\rm{out}}}}\ = \ 10\ {\rm{meV}}$, ${V_{{\rm{in}}}} = \ 40\ {\rm{meV}}$, we can solve the eigenvalues of the hybrid system (see Supplementary Note 2 for details). As shown in Fig. 4b and c, the red shifts and dispersions of the modes are successfully reproduced. Remarkably, we found that the linear red-shift behavior of the P^′^ mode is a hallmark of the plasmon-induced intermolecular interaction. As shown in Fig. 4d, we calculated the energy shift of P^′^ mode with the assumption that the intermolecular-interaction strength depends (purple curves) or does not depend (red and blue curves) on molecular density. Only the ${V_{{\rm{int}}}} = {V_{{\rm{inter}}}}\ \sqrt \rho $ case gives the correct linear energy shift as the function of $\sqrt \rho $, which reveals the coherent intermolecular-interaction nature mediated by plasmons. In addition, this intermolecular-interaction strength can be reflected by the slope of the energy shift of the P^′^ mode, as shown in Supplementary Fig. 8. For stronger interaction strength ${V_{{\rm{inter}}}}$, the P^′^ mode red shifts more rapidly as the density increases. This interesting effect can be applied to evaluate the intermolecular-interaction strength at the nanoscale. Although the coupled-harmonic-oscillators model successfully predicts the energy-shift behaviors of the three modes, it cannot be used to predict the criteria of the appearance of the P^′^ mode. Further work on extending the model to quantitatively predict the triggering condition of the P^′^ mode is promising.

**Figure 5.**
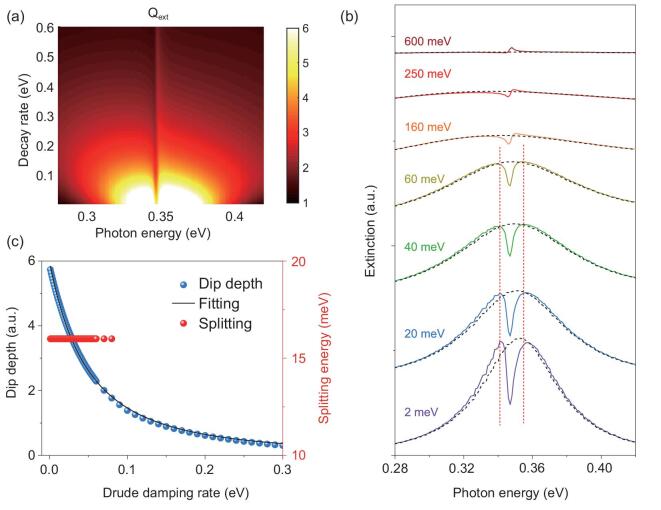
Damping rate-dependent spectral shapes. (a) Extinction efficiency of the SiO_2_@AP@ML hybrid system with various damping rates of the artificial plasmonic (AP) nanoshells. The significant transition of the hybrid modes can be observed. (b) Typical extinction spectra for different damping rates. The extinction spectra of the AP nanoshells are shown in dashed curves for comparison. (c) Dip depth (blue dots) and energy splitting (red dots) as a function of the damping rate of the AP materials, extracted from panel (a).

Finally, we further showed that the damping rate of the plasmonic substrate also impacts the lineshapes of SEIRA, although the molecule–plasmon coupling strength remains unchanged. For metals in the mid-infrared spectral range, the optical loss mainly originates from the intraband transitions, which is depicted by the Drude damping terms in the Drude dielectric model. Experimentally, although it is usually difficult to tune the Drude loss in traditional metals, one can consider the vast candidates of mid-infrared plasmonic materials such as doped semiconductors or graphene to achieve the loss tunings [46,47]. In this work, we calculate the extinction of the hybrid system SiO_2_@AP@ML with the tuning of the Drude damping rate $\gamma $ from 2 to 600 meV of artificial plasmonic (AP) materials and the spectra are shown in Fig. 5a and b. For comparison, the plasmonic background from the pure substrate without coupling to the molecules is shown by the black dashed curves in Fig. 5b. As the damping rate $\gamma $ increases, the depth of the peak split-induced dip gradually decreases. This result is expected because the local field enhancement is inversely proportional to the loss [48,49], with enhancement factor ${\rm{EF}} \propto \frac{Q}{{\sqrt V }}$, where$\ V$ is the mode volume, and $Q = \frac{\omega }{{\Delta \omega }}\sim\frac{\omega }{\gamma }$, where $\omega $ and $\Delta \omega $ are the resonance frequency and linewidth of the mode, respectively [48]. As $\gamma $ increases, the field enhancement decreases and thus the absorption intensities decrease. The summarized dip depth as a function of the damping rate is shown in Fig. 5c, which perfectly matches the theoretical fittings. However, the decrease in field enhancement is not reflected in the peak splitting. Instead, a nearly constant splitting of 16 meV is observed for $\gamma $ varying from 2 to 80 meV, as illustrated by the vertical dashed lines in Fig. 5b. The loss-independent peak splitting indicates that the coupling strength remains unchanged with larger Drude damping because the molecule–plasmon coupling strength is given by ${ g}\ = {\mu _{\rm{m}}}\ | {{E_{{\rm{vac}}}}} |$, where ${\mu _m}$ is the effective transition dipole of the molecular ensemble and ${E_{\rm{vac}}} \propto \frac{1}{{\sqrt V }}$ is the vacuum field related to the local density of the photonic states [48,50]. In the first-order approximation, the mode volume $V\ $does not depend on the material loss [48]. As a result, the coupling strength and the peak splitting are independent of the damping rate [51]. The spectral lineshape still undergoes evolution as the damping rate increases. This evolution can be understood by the analytical model in Eqs (1)–(3), where the asymmetry factor *q* is inversely proportional to ${\mu _{\rm{p}}}\sqrt {{\rho _{\rm{p}}}\hbar { g}}$. The ${\mu _{\rm{p}}}\sqrt {{\rho _{\rm{p}}}} $ term, which reflects the plasmon resonance intensity, scales down with increased damping rate, although $\hbar { g}$ is independent of the damping rate. Therefore, the spectra evolve to the Fano lineshape as the *q* factor increases. An intuitive picture considers the figure of merit for couplings for which the ratio between the coupling strength and the system loss ${ g}/\gamma $ scales down with increased loss. Therefore, the system asymptotically tends toward the weak-coupling regime with the Fano resonance dominating in the spectral features.

## CONCLUSION

In conclusion, we studied the molecule–plasmon interactions and their spectral features based on a multilayered nanosphere model system according to analytical electrodynamic simulation and theoretical models. We show that, even if the plasmon energy is resonant with the molecular vibrations, the spectral profiles can be tuned by the molecule–plasmonic structure distance, the molecular density and the plasmonic damping rate. The detailed derivations show that the molecule–plasmon coupling strength plays a crucial role in determining the spectral lineshapes. For high molecular density, a new resonant mode is observed and is attributed to the plasmon-mediated intermolecular interactions. We prospect that the energy shift of the new mode can be applied to evaluate the nanoscale intermolecular-interaction strength in the plasmonic cavities. This work paves the way toward controllable Fano interference at the nanoscale and more studies on plasmon-dressed molecular electronic or vibrational excited states in various coupling-strength regimes.

## Supplementary Material

nwaa054_Supplemental_File
