# Supplementary material for: Unveiling the molecule–plasmon interactions in surface-enhanced infrared absorption spectroscopy
Source: Natl Sci Rev. 2020 Apr 2;7(7):1228–38. doi: 10.1093/nsr/nwaa054 (PMC8288858; doi:10.1093/nsr/nwaa054)
Supplement: nwaa054_Supplemental_File [file nwaa054_supplemental_file.docx]

Supplementary Material

**Unveiling the molecule-plasmon interactions in surface-enhanced infrared absorption spectroscopy**

Jun Yi^† ,ǁ^, En-Ming You^†^, Song-Yuan Ding^†,*^, and Zhong-Qun Tian^†^

^†^State Key Laboratory of Physical Chemistry of Solid Surfaces (PCOSS), Collaborative Innovation Centre of Chemistry for Energy Materials (iChEM), and Department of Chemistry, College of Chemistry and Chemical Engineering, Xiamen University, Xiamen 361005, China.

*^ǁ^* Jun Yi’s present address is NSF Nanoscale Science and Engineering Center, University of California, Berkeley, CA 94720, USA.

*^*^*Corresponding authors: E-mails: syding@xmu.edu.cn

1. **Multilayer Mie theory**

The analytic solution to the light scattering of a multilayer sphere is solved by the open source code *scattnlay Mie* developed by O. Pena *et al*.[1, 2] according to the improved recursive algorithm of the multilayer Mie theory which is effective and accurate for a wide range of size parameters [3]. The metal shell is described by the Drude model with the following:

$$\varepsilon\left( \omega\right)=\varepsilon_{\text{∞,metal}}-\frac{\omega_{\text{p,metal}}^{2}}{\left( \omega^{2}+i\gamma\omega\right)} (1)$$

where $\varepsilon_{\text{∞,metal}}=27.09$ is the dielectric background at high frequency of gold, $\omega_{\text{p,metal}}=9.33 eV$ is the bulk plasma frequency, and $\gamma=0.068 eV$ is the Drude damping rate.

The ultrathin molecular layer (ML) is described by the Lorentzian model with the following:

$$\varepsilon\left( \omega\right)=\varepsilon_{\text{∞,m}}-\frac{{f_{\text{0,m}}\omega}_{\text{0,m}}^{2}}{\omega^{2}-\omega_{\text{0,m}}^{2}+i\gamma_{\text{m}}\omega} (2)$$

where $\varepsilon_{\text{∞,m}}=1$, $f_{\text{0,m}}=4$ is the effective oscillation strength, $\omega_{\text{0,m}}=0.347 eV$ is the molecular vibrational energy, and $\gamma_{\text{m}}=2 meV$ is the damping rate of molecular vibrations.

The simulated results in the main text are based on Mie calculating of the core-shell particle with SiO_2_@Au@ML unless otherwise stated. The outermost environment is set to be air with refractive index n = 1. For the distance-dependent results as in Figure 2, we add an additional air layer between Au and molecular shell as the spacer layer (namely, SiO_2_@Au@Air@ML). For the density-dependent results as in Figure 3, we modified the dielectric index of the molecules *via* tuning the $f_{\text{0,m}}$ term, as the oscillation strength of the molecular oscillator is linearly proportional to the molecular density. For the decay rate-dependent results as in Figure 4, we modified the dielectric index of the metal layer *via* tuning the $\gamma$ term in the Drude model.

1. **Coupled harmonic oscillator model**

Following the coupled harmonic oscillators model for the polariton dispersion,[4] the anti-crossing feature and the dispersion of the hybrid states are fitted as follows:

$$\hbar\left( \begin{matrix} \omega_{\text{m}} & g \\ g & \omega_{\text{p}} \end{matrix} \right)\left( \begin{matrix} \alpha\\ \beta\end{matrix} \right)=E\left( \begin{matrix} \alpha\\ \beta\end{matrix} \right) (3)$$

where ${\hbar\omega}_{\text{m}}$ and ${\hbar\omega}_{\text{p}}$are the respective resonance energies of the molecules and plasmonic structures, $\hbar g$ is the coupling strength, *E* is the diagonal matrix with the eigenvalues of the coupled states, and $\alpha$ and $\beta$ are the eigenvectors. The eigenvalues are solved as follows:

$$E_{\pm}=\left( \frac{{\hbar\omega}_{\text{m}}+{\hbar\omega}_{\text{p}}}{2} \right)\pm\sqrt{{(\hbar g)}^{2}+\frac{1}{4}{({\hbar\omega}_{\text{m}}-{\hbar\omega}_{\text{p}})}^{2}} (4)$$

The energy detuning is defined as $\delta={\hbar\omega}_{\text{m}}-{\hbar\omega}_{\text{p}}$. Under the zero-detuning condition, the energy splitting of the modes is $\hbar\Omega=E_{+}-E_{-}=2\hbar g$ according to equation (4).To consider the plasmon-mediated intermolecular interaction in the coupled harmonic model, it’s necessary to consider three oscillators coupling in the hybrid system. In the studied system, the molecules in the molecular shell locate at various positions such as inside the hotspots and outside the hotspots for simplicity, and experience different coupling strength with the plasmons. The molecules inside the hotspot and those outside the hotspots can be simplified as two kinds of oscillators that coherently coupled with plasmon with various coupling strength, as shown in Figure 4a in the main text, the $V_{i}$ and $V_{o}$ terms. Due to the coherent coupling, the molecules at the two locations are indirectly coupled through the plasmonic field, and phenomenally leading to long-range intermolecular interactions that are mediated by plasmons ($V_{\mathrm{int}}$ term). The Hamiltonian of the interacting system is given by

$$\mathbf{H}=\left( \begin{matrix} \hbar\omega_{m,out} & V_{\mathrm{int}} & V_{o} \\ V_{\mathrm{int}} & \hbar\omega_{m,in} & V_{i} \\ V_{o} & V_{i} & \hbar\omega_{p} \end{matrix} \right) \left( 5 \right),$$

where $\hbar\omega_{m,out}$, $\hbar\omega_{m,in}$ and $\hbar\omega_{p}$ are the vibrational energies of the molecules outside the hotspots and inside the hotspots, and resonance energy of plasmonic structure, respectively. Under the zero-detuning condition, $\hbar\omega_{m,out}=\hbar\omega_{m,in}=\hbar\omega_{m,p}$. The $V_{\mathrm{int}}$, $V_{o}$ and $V_{i}$ are the plasmon-mediated intermolecular coupling strength, the molecule-plasmon coupling strength of molecules outsider hotspots, and that of molecules insider hotspots, respectively. The molecule-plasmon coupling strength $V_{i}$ and $V_{o}$ would scale up with the square root of the molecular density$\sqrt{\rho}$, as elaborated previously. Here we define the $V_{i}\equiv V_{\mathrm{in}}\sqrt{\rho}$ and $V_{o}\equiv V_{\mathrm{out}}\sqrt{\rho}$ to clarify the scaling. Since the coupling strength depends on the local photonic density of state as we discussed above, it thus requires $V_{\mathrm{in}}>V_{\mathrm{out}}$. For the plasmon-mediated intermolecular coupling term $V_{\mathrm{int}}$, we phenomenally assume it also scale up with $\sqrt{\rho}$ and define $V_{\mathrm{int}}\equiv V_{\mathrm{inter}}\sqrt{\rho}$ since the plasmon induced intermolecular interaction is mainly dominated by the interactions between plasmon and molecules outside the hotspots.

The mode energy of the coupled system can be solved via the equation $H\varphi=E\varphi$, where *E* is the eigenvalue of the system and $\varphi$ is the eigenvector. The eigenvalue *E* thus can be solved *via* the determinant of the matrix as det(*H-IE*) = 0.

1. **Theoretical model for molecule-plasmon coupling in weak-coupling regime**

In this work, we used a simple quantum mechanical model[5-7] to describe the Fano interference between the molecular vibrational mode and plasmonic mode for further analysis of the Mie calculation results. We consider the molecule as a two-level system with a ground state $|\left. 0 \right\rangle$, and vibrational excited state $|\left. e \right\rangle$ which interact with a continuum set of plasmon states $|\left. P \right\rangle$. The schematics are shown in Fig. S3.

The Hamiltonian of the system is given by the following:[5-7]

$$\hat{H}=\hat{H}_{m}+\hat{H}_{p}+\hat{V}={\hbar\omega}_{m}\left| \left. e \right\rangle\right.\left. \left\langle e \right. \right|+\int d\omega\hbar\omega\left| \left. P_{\omega} \right\rangle\right.\left. \left\langle P_{\omega} \right. \right|+\int d\omega V_{\omega}\left| \left. P_{\omega} \right\rangle\right.\left. \left\langle e \right. \right| \left( 6 \right),$$

where ${E_{m}=\hbar\omega}_{m}$ is the energy of the molecular vibrational excited state. We define the energy of the ground state $\left| \left. 0 \right\rangle\right.$ as zero. The plasmonic states are nondegenerate and orthogonal, which gives $\left\langle P_{\omega} | \hat{H} | P_{\omega'} \right\rangle=\hbar\omega'\delta(\omega-\omega')$; $V_{\omega}$ is the matrix element of the interacting Hamiltonian $\hat{V}$; and the interaction strength of the system is described as $\hbar g(\omega)={|V_{\omega}|}^{2}=\left| \left\langle P_{\omega} | \hat{H} | e \right\rangle\right|^{2}$, which is dependent on the oscillation strength of the molecular dipoles and the plasmon-mediated vacuum field.[6, 8]

The new set of orthogonal eigenstates $\left| \left. \Psi_{\omega} \right\rangle\right.$ of the system with interactions are obtained following Fano’s orthogonalization method, which is given by the following:[5]

$$|\Psi_{\omega}> = \frac{sin\Delta}{\sqrt{\pi\gamma\left( \omega\right)}}|\Phi_{\omega}>-cos\Delta|P_{\omega}> (7)$$

where $\left| \left. \Phi_{\omega} \right\rangle\right.=\left| \left. e \right\rangle\right.+P.V.\int d\omega'\frac{V_{\omega}}{\hbar\left( \omega-\omega' \right)}\left| \left. P_{\omega'} \right\rangle\right.$ is considered as the dressed excited state $\left| \left. e \right\rangle\right.$ due to the molecule-plasmon coupling, and $P.V.$ indicates the Cauchy principal value of the integral. Additionally, $\Delta$ is the phase shift induced by the Fano interference between the excited state $\left| \left. e \right\rangle\right.$ and plasmon states $\left| \left. P_{\omega} \right\rangle\right.$, and $\gamma\left( \omega\right)$ describes the modified linewidth the eigenstates, and they are given as follows:

$$\gamma\left( \omega\right)={\pi|V_{\omega}|}^{2}=\pi\hbar g\left( \omega\right) (8)$$

$$\Delta\left( \omega\right)=-\arctan\frac{\gamma\left( \omega\right)}{\hbar\omega-\hbar\omega_{m}-F\left( \omega\right)} (9)$$

The phase shift $\Delta\left( \omega\right)$ varies by $\sim\pi$ as $\omega$ traverses an interval of $\gamma\left( \omega\right)$ around the resonance energy of $\hbar\omega_{m}+F(\omega)$, and thus $F(\omega)$ is considered as the energy shift of the excited state $\left| \left. e \right\rangle\right.$ induced by the coupling, which is given by the following:

$$F\left( \omega\right)=P.V.\int d\omega'\frac{{|V_{\omega}|}^{2}}{\hbar(\omega-\omega')} (10)$$

The absorption rate of photons with energy $\hbar\omega$ by the system can be represented as the squared matrix element of a transition operator $\hat{T}$ from the ground state $\left| \left. 0 \right\rangle\right.$ to the hybrid excited state $\left| \left. \Psi_{\omega} \right\rangle\right.$, which is given by Fermi’s gold rule as follows:

$$W_{0\to\Psi_{\omega}}=\frac{2\pi}{\hbar}\int d\omega^{'}\left| \left\langle\Psi_{\omega} | \hat{T} | 0 \right\rangle\right|^{2}\delta\left( \omega-\omega^{'} \right) (11)$$

Therefore, the absorption of the coupled system is written as follows:

$$A\left( \omega\right)=n_{p}\hbar\omega W_{0\to\Psi_{\omega}}=2\pi n_{p}\omega\left| \left\langle\text{Ψ}_{\text{ω}} | \hat{T} | 0 \right\rangle\right|^{2} (12)$$

where $n_{p}$ is the number of photons per area per second, which is proportional to the power density of the light. By substituting the formula of $\Psi_{\omega}$ in equation (7) into equation (12) and by defining a dimensionless parameter $\epsilon=-\cot\Delta$, the Fano absorption profile is obtained as follows:

$$A\left( \omega\right)=2\pi n_{p}\omega\left| \left\langle P_{\omega} | \hat{T} | 0 \right\rangle\right|^{2}\frac{{(q+\epsilon)}^{2}}{\epsilon^{2}+1} (13)$$

where the last term $\frac{{(q+\epsilon)}^{2}}{\epsilon^{2}+1}$ is the Fano function, which depends on an asymmetry factor $q$ and the reduced energy $\epsilon$, and the second term $\left| \left\langle P_{\omega} | \hat{T} | 0 \right\rangle\right|^{2}$ is the absorption of the uncoupled plasmonic cavity, which is considered as the broad background in the absorption spectra. The asymmetry factor $q$ is given by the following:

$$q=\frac{\left\langle\text{Φ}_{\omega} | \hat{T} | 0 \right\rangle}{\sqrt{\pi\gamma\left( \omega\right)}\left\langle P_{\omega} | \hat{T} | 0 \right\rangle} (14)$$

which is proportional to the transition rate to the dressed excited states $\left| \left. \Phi_{\omega} \right\rangle\right.$ and to the uncoupled plasmonic states.

One can find that the interaction term $V_{\omega}$ critically determines the properties of the hybrid system, and it highly depends on the vacuum field mediated by the plasmons, which is connected with the density of plasmonic states as $E_{\mathrm{vac}}(\boldsymbol{r},\omega)\propto\sqrt{\rho_{p}(\boldsymbol{r},\omega)}$, where $\rho_{p}\left( \boldsymbol{r},\omega\right)$ is the normalized density distribution of the photonic states such as plasmonic states [8].

Although the spatial distribution of $\rho_{p}(\boldsymbol{r},\omega)$ is generally difficult to evaluate from the experimental data, the overall variation of $\rho_{p}(\boldsymbol{r},\omega)$ as a function of frequency, can be approximately extracted from the far-field spectra as $\rho_{p}(\boldsymbol{r},\omega)\propto C_{\mathrm{ext}}(\omega)$, where $C_{\mathrm{ext}}(\omega)$ is the normalized extinction cross-section. To obtain the analytical forms of $q$ and $\epsilon$, we simplify the density of states in terms of a Lorentzian profile as a function of frequency centered at the plasmon resonance frequency $\omega_{p}$ with linewidth $r_{p}$. This assumption is valid if only one plasmon mode is interacting with the molecules.

$$\rho_{p}\left( \omega\right)=\frac{1}{1+{(\frac{\hbar\omega-{\hbar\omega}_{p}}{r_{p}/2})}^{2}} (15)$$

In this case, the coupling strength $\hbar g\left( \omega\right)=\hbar g_{0}\rho_{p}\left( \omega\right)$, with $\hbar g_{0}=v^{2}$ and the maximal coupling strength at the plasmon resonance ${\hbar\omega}_{p}$. We define the transition moment from the ground state to the uncoupled vibrational excited state $\left| \left. e \right\rangle\right.$ as a transition matrix element $t_{m}=\left\langle e | \hat{T} | 0 \right\rangle$ and the transition moment from the ground state to the uncoupled plasmonic state $\left| \left. P_{\omega} \right\rangle\right.$ as $\left\langle P_{\omega} | \hat{T} | 0 \right\rangle=\mu_{p}\sqrt{\rho_{p}\left( \omega\right)}$. By substituting equation (15) into equation (7) and (10), the principal value of the integral is solved, where the energy shift is written explicitly as $F\left( \omega\right)=\pi hg(\omega)\frac{\hbar\omega-\hbar\omega_{p}}{\gamma_{p}}$, and the absorption is derived as follows:

$$A\left( \omega\right)=2\pi n_{p}\omega\mu_{p}^{2}\rho_{\text{p}}\left( \omega\right)\frac{\left( q+\epsilon\right)^{2}}{\epsilon^{2}+1} (16)$$

where the asymmetry factor $q$and reduced energy $\epsilon$ are as follows:

$$q=\frac{t_{m}}{\pi\mu_{p}\sqrt{\rho_{\text{p}}\hbar g}}+\frac{\hbar\omega-\hbar\omega_{p}}{\gamma_{p}/2} \left( 17 \right)$$

$$\epsilon=\frac{\hbar\omega-\hbar\omega_{m}}{\pi\hbar g}-\frac{\hbar\omega-\hbar\omega_{p}}{\gamma_{p}} (18)$$

Therefore, the coupling strength critically determines the Fano parameters and the Fano spectral profiles.

For the model results as in Figure 2c and 2d, the $t_{m}$ and $\mu_{p}$ are approximately estimated via the linewidth of the uncoupled molecular resonance and plasmon resonance, respectively. Here we set $t_{m}$ to be 1.6 meV and $\mu_{p}$ to be 15.9 $\sqrt{\mathrm{meV}}$. The coupling strength $\hbar g\left( \omega\right)=\hbar g_{0}\rho_{p}\left( \omega\right)$ is a function of frequency with $\rho_{p}\left( \omega\right)$ the density of states. The $\hbar g_{0}$ thus is the only input parameter which we obtained via fitting the simulation results at the same condition, as shown in the Figure 2b.

With these parameters, for each coupling strength $\hbar g_{0}$, we can directly calculate the Fano parameters $q$ and $\epsilon$ as functions of frequency $\omega$, as well as the extinction spectra using the equation (16). We note that for simplicity the distance decay induced absorption intensity attenuation is not included in our model, and the absolute extinction intensities of the spectra are mainly determined by the $C_{\mathrm{ext}}(\omega)$ term, which is equivalent to normalization of the absorption intensity.


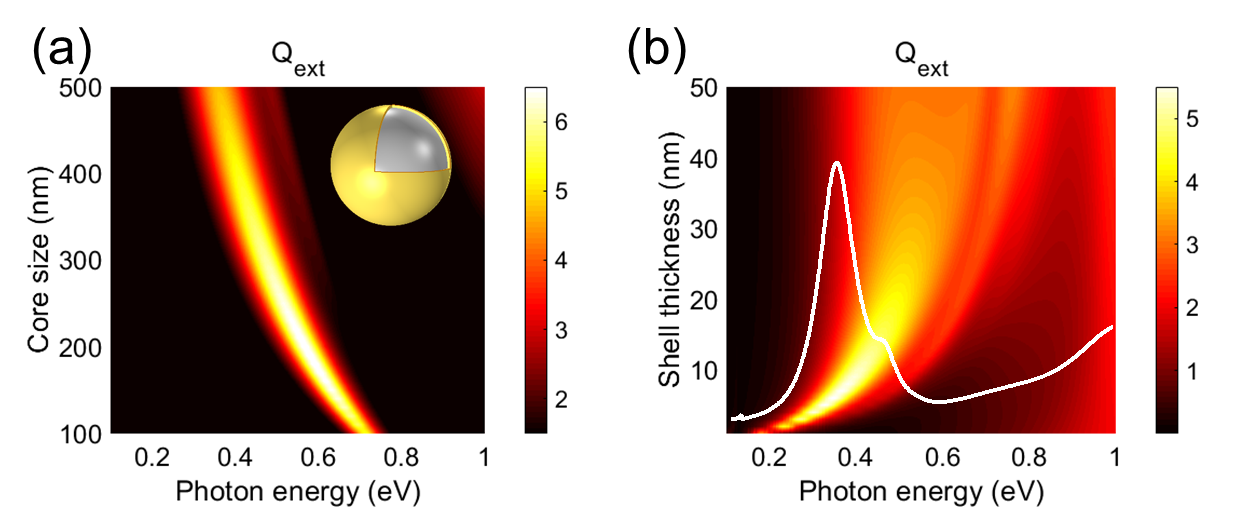


**Figure S1.** Optical properties of a SiO_2_@Au core-shell particle. (a) Extinction efficiency spectra maps as a function of the core radius with fixed shell thickness at 10 nm. The particle geometry is shown in the inset. The resonance energy redshifts as the SiO_2_ core size increases. (b) Extinction spectra map as a function of the shell thickness with fixed core radius at 400 nm. We select the particle with a core size of 400 nm and a shell thickness of 6 nm as a typical plasmonic nanoshell structure. The corresponding extinction spectrum is shown in white curves in (b).


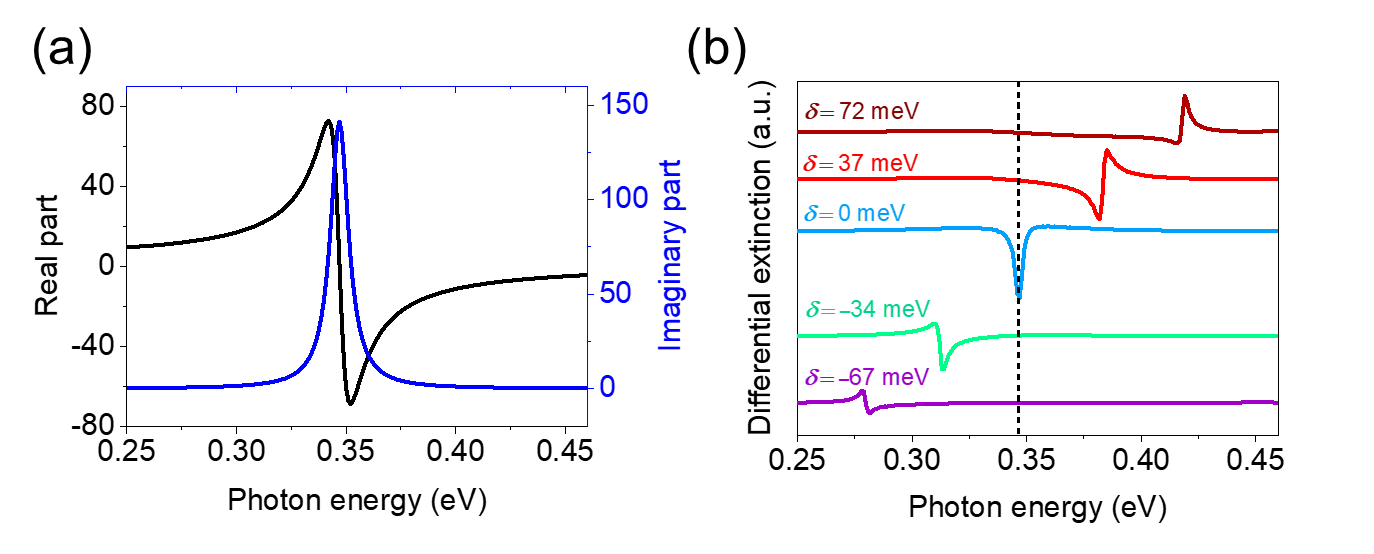


**Figure S2.** Optical response of uncoupled and coupled molecules. (a) The real and imaginary part of the dielectric function of uncoupled molecular layers are described by the Lorentzian model. (b) Differential extinction spectra are obtained by subtracting the extinction spectra of the plasmonic structures (uncoupled system) from those of the molecule-plasmon coupling system. Different detuning energies by changing the molecular vibrational energy are labeled on the spectra.


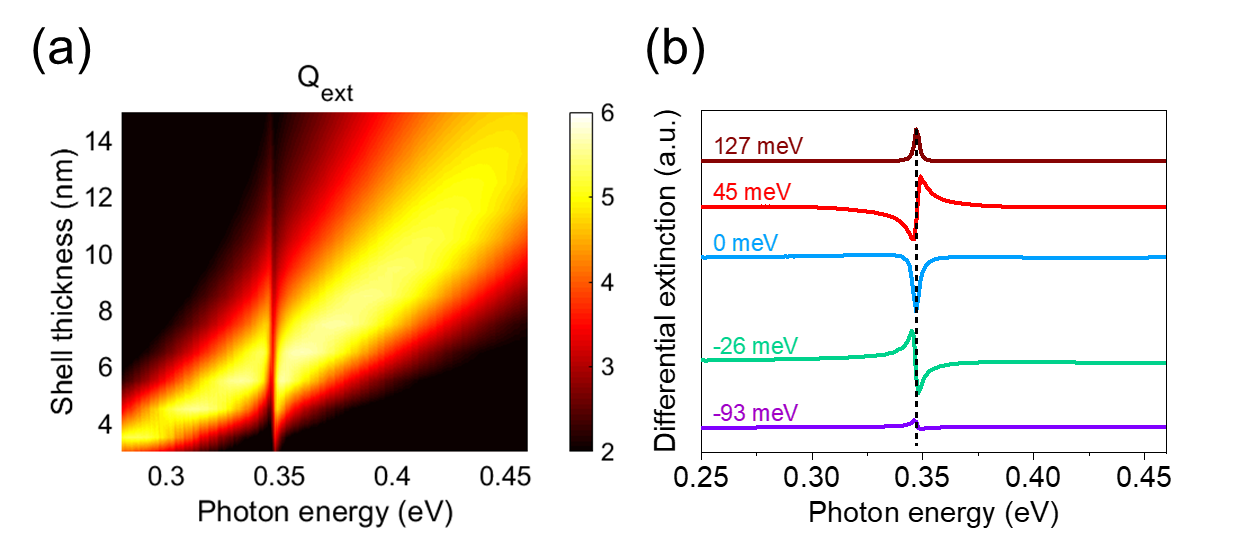


**Figure S3.** Optical properties of molecules-plasmon hybrid system under various energy detuning. (a) Extinction efficiency spectra of the molecular layer coupled with the SiO_2_@Au particle with resonance energy varied by changing the shell thickness of the Au shell (indicated in the vertical axis). The vibrational resonance of the molecules is fixed at 0.347 eV, and the detuning $\delta$ varies from −0.12 eV to 0.15 eV. (b) Typical detuning-dependent differential extinction spectra of molecules.


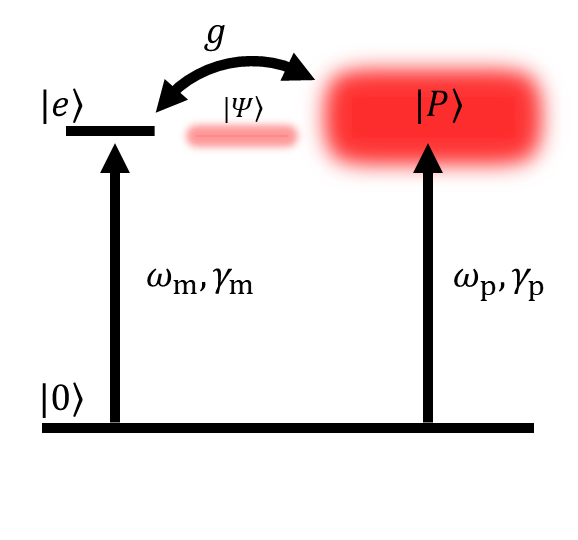


**Figure S4.** Sketch of Fano interference between molecules and plasmons. The $\left| \left. 0 \right\rangle\right.$, $\left| \left. e \right\rangle\right.$, and $\left| \left. P \right\rangle\right.$ denote the vibrational ground states and excited states of the molecules and the excited states of plasmons, respectively. The transition energy and decay rate of molecular excitation are $\omega_{m}$ and $\gamma_{m}$, respectively, and $\omega_{p}$ and $\gamma_{p}$ represent the transition energy and damping of plasmons, respectively. The vibrational excited states of the molecules interact with the plasmons with coupling strength $\hbar g$. A new hybrid eigenstate $\left| \left. \Psi\right\rangle\right.$ is formed due to the coupling.[7]


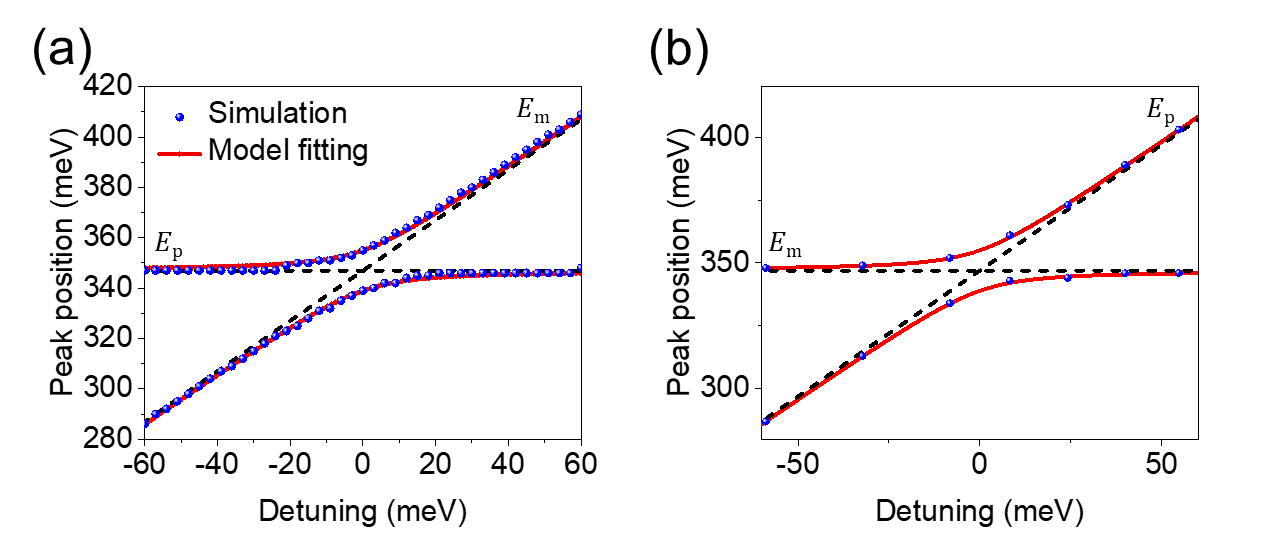


**Figure S5.** Dispersion of molecule-plasmon hybridizations. (a) Dispersions extracted from Fig. 1b with varying molecular vibrational energy. (b) Dispersions extracted from Fig. S3 with varying plasmon resonance energy. The blue dots are extracted from the spectral peaks, which represent the energies of hybrid modes. The dispersion clearly shows the anti-crossing features. The extracted mode energies are fitted by a coupled oscillator model, as shown in the red solid curves. In (a), the plasmon-resonance energy remains at 347 meV, and the phonon energy of molecules varies from 280 meV to 410 meV; For (b), the phonon energy of molecules remains at 347 meV and the plasmon-resonance energy varies from 280 meV to 410 meV. The uncoupled molecular vibrational energy and plasmon energy are indicated by the dashed lines and are labeled as $E_{m}$ and $E_{p}$, respectively. The splitting energy in the zero-detuning condition is 16.1 meV, which is smaller than the decay rates of the plasmons, and thus the coupling remains in the weak-coupling regime.


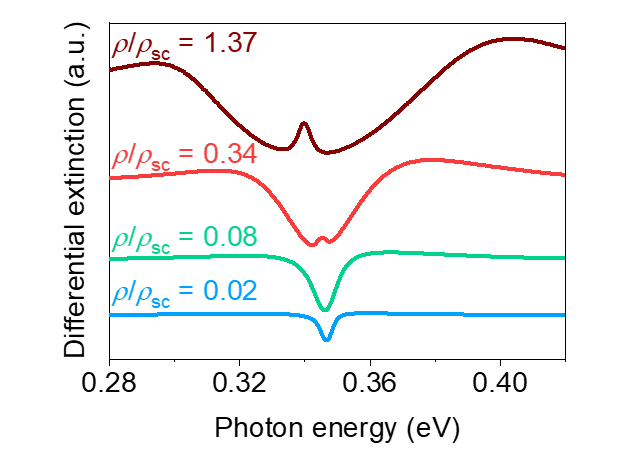


**Figure S6.** Typical differential extinction spectra with relative molecular density $\rho/\rho_{\mathrm{sc}}$ varied from 0.02 to 1.37. As the relative density increases, the anti-resonance dip deepens, indicating the stronger coupling between molecules and plasmons. When $\rho/\rho_{\mathrm{sc}}>0.34$, a new resonance peak near 0.35 eV is observed. This mode is enhanced and redshifted to lower energy with larger molecule density.


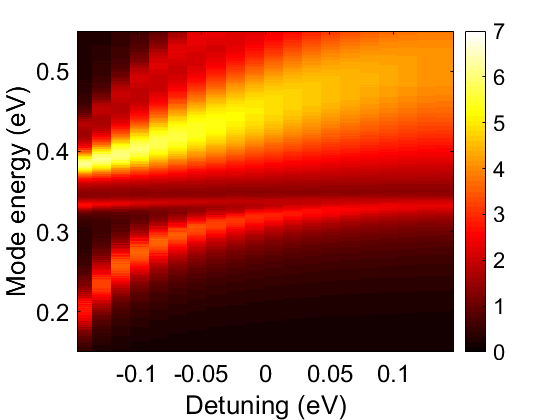


**Figure S7.** Extinction efficiency maps of the hybrid system with $\rho/\rho_{\mathrm{sc}}=2$ (Rabi splitting ~120 meV). The plasmon energy is tuned by changing the shell thickness.


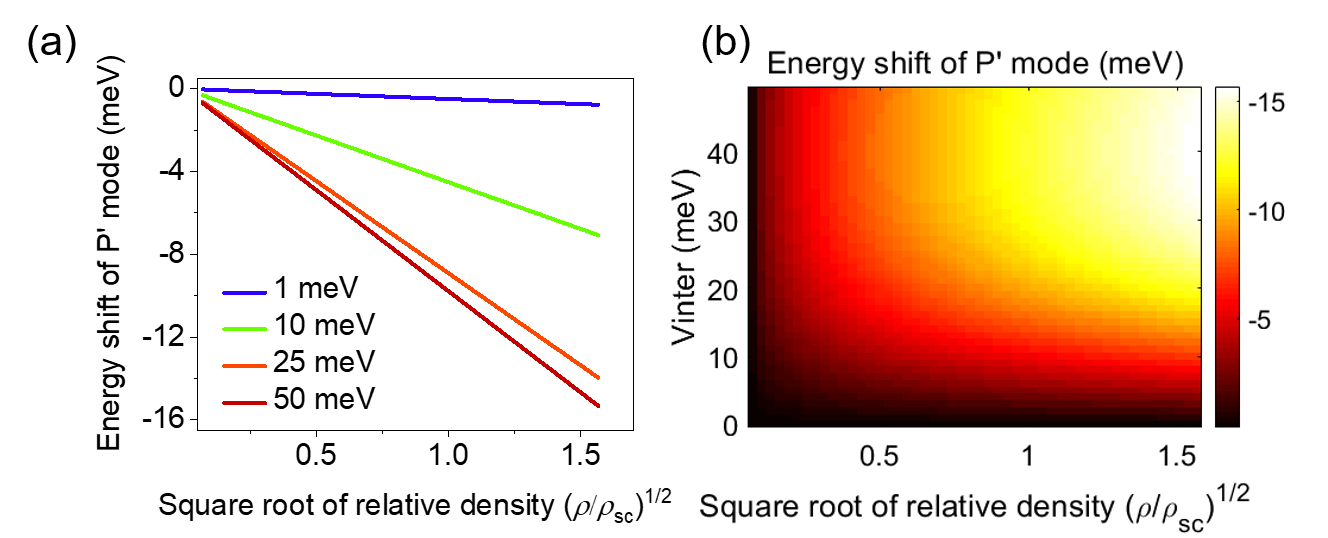


**Figure S8.** The intermolecular interaction strength-dependent energy shift of P' mode. (a) Energy shift of P' mode as a function of the square root of relative molecular density, with various intermolecular interaction strength $V_{\mathrm{inter}}$ from 1 to 50 meV. For larger $V_{\mathrm{inter}}$, the slope of energy shift become larger. (b) Maps of the energy shift of P' mode as a function of the square root of relative molecular density and $V_{\mathrm{inter}}$. For larger density or $V_{\mathrm{inter}}$, the energy shift would be increase. The shifted energy of P' mode thus reflects the plasmon-induced intermolecular interaction strength.

**REFERENCES**

1. Peña O and Pal U. Scattering of electromagnetic radiation by a multilayered sphere. *Comput Phys Commun* 2009; **180**: 2348-54.

2. Ladutenko K, Pal U, Rivera A*, et al.* Mie calculation of electromagnetic near-field for a multilayered sphere. *Comput Phys Commun* 2017; **214**: 225-30.

3. Yang W. Improved recursive algorithm for light scattering by a multilayered sphere. *Appl Opt* 2003; **42**: 1710-20.

4. Deng H, Haug H and Yamamoto Y. Exciton-polariton Bose-Einstein condensation. *Rev Mod Phys* 2010; **82**: 1489-537.

5. Fano U. Effects of Configuration Interaction on Intensities and Phase Shifts. *Phys Rev* 1961; **124**: 1866-78.

6. Osley EJ, Biris CG, Thompson PG*, et al.* Fano Resonance Resulting from a Tunable Interaction between Molecular Vibrational Modes and a Double Continuum of a Plasmonic Metamolecule. *Phys Rev Lett* 2013; **110**: 087402.

7. Giannini V, Francescato Y, Amrania H*, et al.* Fano Resonances in Nanoscale Plasmonic Systems: A Parameter-Free Modeling Approach. *Nano Lett* 2011; **11**: 2835-40.

8. Giannini V, Fernández-Domínguez AI, Heck SC*, et al.* Plasmonic Nanoantennas: Fundamentals and Their Use in Controlling the Radiative Properties of Nanoemitters. *Chem Rev* 2011; **111**: 3888-912.
